# A New Tool for Determining and Monitoring Public Healthcare Systems

**DOI:** 10.3390/healthcare10122528

**Published:** 2022-12-14

**Authors:** Vered Reiter, Doron Nisani, Shay S. Tzafrir, Nathaniel Laor

**Affiliations:** 1 Association for Children at Risk, Center for the Study of Organizations & Human Resource Management, University of Haifa, Haifa 3498838, Israel; 2 School of Business Administration, University of Haifa, 199 Abba-Khoushy Ave, Mount Carmel, Haifa 3498838, Israel; 3 School of Business Administration, Center for the Study of Organizations & Human Resource Management, University of Haifa, Haifa 3498838, Israel; 4 Department of Psychiatry, Sackler Faculty of Medicine, Tel Aviv University, Tel Aviv 6195001, Israel; 5 The Child Study Center, Yale University, New Haven, CT 06520, USA

**Keywords:** nationalized healthcare, stakeholders, reasonable standards, public health services

## Abstract

The challenge of maintaining a standard of treatment has become a core issue due to the COVID-19 outbreak, and many countries are currently addressing this issue. Since public health policymaking is a multidimensional issue, including different aspects, measures, features, and scales, and so forth, multidimensional definitions of reasonable medical treatments may improve planning and performance standards for public health systems. This study emphasizes the need to settle all of the dimensions in policymaking to aim to elicit reasonable medical treatment definitions and adequacy assessments from diverse healthcare stakeholders and offer a universally applicable reasonable medical treatment formula. Interviews of thirty-two stakeholders were qualitatively analyzed and mapped onto an innovative quadrilateral model. The findings showed that most interviewees viewed the system positively. However, they identified various lacunas—clinical/service, social/ethical, legal, and economically reasonable medical treatment aspects. A generic formula for the medical sub-services’ activity accounted for these, given any specific time period and technological development. The stakeholders’ positive assessment reflects an acquiescence for resource allocation and policy enforcement, rather than optimal healthcare. Nationally, this should be addressed. The quadrilateral mapping of the stakeholders enhances the translatability and generalizability of the systemic data. A comprehensive reasonable medical treatment formula will help the policymakers to optimize services, and it will render healthcare planning/implementation transparent, effective, and responsible.

## 1. Introduction

The human cost of the COVID-19 outbreak continues to mount, and many countries are implementing very severe steps. People are required to give up some of their liberties, allowing authorities to intrude into their family and social lives under the presumption that it will maintain individual and societal health. Among other consequences, these countries have made challenging decisions in order to fund vaccines for the epidemic. Consequently, budgets were transferred to fund the vaccine and other medical operations caused by the pandemic, and this affected the ongoing budget of the health system, which contains critical factors that will affect the future health of patients. While the leaders and experts deal with monitoring the direct consequences of the pandemic, less attention is being given to the indirect medical treatments on the population.

The national governments in high-income countries with aging populations are concerned with innovatively and effectively meeting the increasing demands for public healthcare services [1]. Public debate on this issue is abound, particularly due to the persistent inequality experienced by the individuals or groups of consumers in such health systems, and it is often determined by implicit social norms, the interplay of administrative and political forces, or explicit standards of reasonable care [2,3]. To advance a responsible societal culture of health improvement for the common good, a multidimensional public health meta-framework offering an abstract formulation of existing Western public healthcare systems [4], such as the UK National Health Service (UK Web Archive (accessed on 4 April 2015), National Care Standards, https://www.webarchive.org.uk/wayback/archive/20150404100647/http:/www.nationalcarestandards.org/ (accessed on 4 April 2015)), the US Patient Protection and Affordable Care Act (U.S. Department of Health and Human Services. (n.d.), Patient Protection and Affordable Care Act, Healthcare.Gov, https://www.healthcare.gov/glossary/patient-protection-and-affordable-care-act/ (accessed on 4 April 2015)), and the World Health Organization (WHO Essential Medicines and Health Products Department, Essential medicines and health products, World Health Organization, 2020), was recommended. It was suggested that such a formulation should relate to a range of domains [4] (from ecology to biomedical science), levels (from citizen participants to national policymakers), and recommended interventions (spanning urban-structural to individual lifestyle programs).

In line with such recommendations, in order to elucidate a reformulation of the responsible public healthcare services, the present study emphasizes the necessity to refer to as many of the stakeholders within the health system as possible, and it aims to define operationally “reasonable” care. This formula is in fact a tool to help the policymakers to organize, prioritize, and optimize the policies that they determine. As a case study for exploring healthcare “reasonability”, we examined the case of Israel’s health system, following the legislation in 1995 which required the medical community to provide all of the citizens with “reasonable medical treatment” (RMT). Utilizing a qualitative methodology, the current study aimed to elicit the definitions and formulations of RMT as voiced by the diverse stakeholders that are internal and external to Israel’s national health insurance system. Specifically, this analysis sought, firstly, to map Israel’s relevant stakeholders’ views and interests concerning medical practice to comply with the legal, systemic, and clinical requirements, and, secondly, to offer a generic formula for the RMT that could be applicable beyond the case of Israel. We thus hope to promote a responsible, open discussion of the possible problems and solutions which are on the way to developing a liberal national health insurance system based on the explicit standards for social action, given the constraints of each country’s ideologies, stakeholder interests, and available systemic resources.

## 2. Materials and Methods

### 2.1. The Israeli Health System Context

Israel’s National Health Insurance Law (NHIL) (Israeli Ministry of Foreign Affairs. (1 September 1995), National Health Insurance, https://mfa.gov.il/mfa/mfa-archive/1990-1995/pages/national%20health%20insurance.aspx#:~:text=The%20National%20Health%20Insurance%20Law,country%20(not%20including%20tourists (accessed on 4 April 2015)) of January 1995 sought to reshape the country’s health system in line with the recommendations of the National Investigation Committee on Health Care in Israel (Knesset, (accessed on 24 October 1990), Report of the Netanyahu Committee on the health system (continued discussion), https://oknesset.org/meetings/2/0/2050145.html (accessed on 4 April 2015)) so that citizens could enjoy an equal entitlement to a basic “health basket” of “reasonable” medical services:

“Health services which are part of the “health basket” will be provided in Israel according to medical considerations, will be of **reasonable** quality, within a **reasonable** period of time and at a **reasonable** distance from the insuree’s residence, all to be funded by the health funds’ resources…. The health basket is a set of services, activities, and goods that are considered to be essential and thus within the scope of reimbursement or direct service delivery”.

The NHIL placed the responsibility for defining the health basket, determining its “reasonable” terms of delivery, and monitoring this process into the hands of the Ministry of Health—the major administrative, professional, and regulatory stakeholder in the healthcare system [5]. The “reasonable” health basket was to be updated periodically by professional committees based on the budgets, new scientific and technological medical knowledge, ethical principles, consumer representative interests, local pharmaceutical considerations, and service providers’ interests. It is to be noted that although the 1995 law prescribed the need for definition of the health basket, it did not interfere in the determination of that basket’s specific requirements or standards, beyond stating that it ought to be reasonable.

Indeed, to date, the Israeli legislature has not yet specified well-defined standards to guide the institutional development, planning, and monitoring of “reasonably” good medical practice offered at the national level. The NHIL’s undefined terms—reasonable quality, reasonable time, and reasonable distance—are susceptible to different interpretations by the health system’s diverse and often competitive stakeholders [6,7,8]. Moreover, since the law has refrained from establishing specific, detailed conceptual and practical parameters related to RMT, it may invite conflicts between the professional, administrative, and political considerations. As a result, the system may be susceptible to bureaucratic ad hoc manipulations coupled with extreme regulations (either too lax or too strict) to guard against, suppress, or facilitate the influence of interested parties and groups, very often at the expense of the healthcare system’s ultimate consumers.

### 2.2. Reasonability As the Standard for Responsible Social Action

Reasonable national medical planning has been justified morally and legally by viewing healthcare as a universal right. However, the health planners must decide how to distribute healthcare universally and equally while allowing for economic constraints. A utopian vision of global unlimited insurance for a whole population is unreasonable because it could inevitably lead to the state’s bankruptcy. In addition, unjust socio-political processes may lead to unreasonable, inadequate healthcare for those in the lower socioeconomic strata or marginalized subgroups [9,10], thereby calling for regulatory intervention that goes beyond the system’s usual boundaries [11]. Yet, regulation requires clinical, ethical, and legal standards of application, particularly when different levels of partnerships may evolve between the public and private healthcare services [11]. The integrated set for these standards of application may be termed RMT. A standard, explicit definition of reasonable medical behavior or a reasonable level of care that is open for public inspection and criticism could enable the healthcare executives and health practitioners to optimize responsible, transparent, high-quality clinical care that permits optimal levels of service (i.e., patient satisfaction) while maintaining the systemic cost-effectiveness that aligns with the pragmatic budgetary constraints. Four main aspects characterize “reasonability” as a standard of responsible action in various social systems—the economic, clinical, legal, and socio-ethical one [12,13]. In medical systems, these four aspects often intertwine.

Optimally, for a healthcare system to operate reasonably and responsibly at each systemic level, all of the participating stakeholders need to maintain a shared viewpoint of what is reasonable [14]. However, this is rarely possible. Instead, the received RMT in a given society could be derived by analyzing the shared and contrasting viewpoints of diverse stakeholders inside and outside of the healthcare system.

### 2.3. Definition and Empirical Analysis of Stakeholders

Although doctors and patients are the core stakeholders in complex healthcare systems, many more interest groups play a role in organizational strategy and decision-making [8]. In Israel, these include: (a) private and public direct service providers, hospitals, and clinics; (b) the senior managers of health “funds” (akin to health maintenance organizations or HMOs), responsible for implementation of health policy; (c) the regulating body (i.e., the Ministry of Health); (d) the citizens/consumers (individuals, communities, populations at risk); (e) the third sector, comprising non-profit healthcare NGOs that serve the public.

Initially, the stakeholder theory [15] considered only the groups inherent to an organization. Later, the definition of stakeholders included “actors who have an interest in the issue under consideration, who are affected by the issue, or who—because of their position—have or could have an active or passive influence on the decision-making and implementation process [8].” The most widely accepted definition [16] is: stakeholders are “persons or groups that have, or claim, ownership, rights, or interests in a corporation and its activities, past, present or future”. These broader systemic views allow for the consideration of stakeholders both within the organization (internal) and in its larger environment (external).

Taking this distinction one step further, Malvey, Fottler, and Slovensky detailed which stakeholders hold “internal” positions in the organizations—such as executive managers, boards of directors, ethics committees, company departments (financial, marketing, human resources, etc.)—as well as which stakeholders hold positions that are “external” to the organizations, such as owners, shareholders, suppliers, consumers, and local/wider community representatives [17]. Moreover, they also defined some individuals as “interface” stakeholders, such as physicians and other clinical service providers [17].

Importantly, Clarkson contributed another major theory on stakeholders, differentiating between the primary and secondary stakeholders of any system of service delivery [16]. The “Primary” stakeholders comprise employees, customers, investors, and suppliers on the one hand, and the public stakeholders represent groups such as the governments and communities that provide infrastructures on the other hand. Thus, organizations cannot survive without the primary stakeholders’ continuing participation. The “Secondary” stakeholders comprise those who influence or are influenced by the organization, but they are not engaged in transactions and are not essential for its survival [18], such as the media and diverse special interest groups.

Though they differ in their emphasis, these theories on internal/external, primary/secondary, and organizational positions provide the best available foundations for stakeholder analyses as they utilize the stakeholders’ management theory in order to illuminate significant challenges in business management as well as in healthcare management. Each group of stakeholders presents different and sometimes conflicting perspectives, experiences, and areas of expertise [14,19]. A stakeholder analysis is an approach or set of tools for generating knowledge about the system’s diverse participants’ behaviors, intentions, interrelationships, and interests to assess the influences they bear on responsible strategic decision-making or implementation processes [8].

### 2.4. Study Objectives

Firstly, the current study aimed to elicit the possible conflictual and complementary viewpoints of the diverse stakeholders who are central players [20,21] in the Israeli healthcare system [12]. We analyzed various stakeholder groups’ perceptions of the reformed (post-NHIL) Israeli healthcare system’s merits and deficits.

Secondly, to render the investigated Israeli stakeholder groups’ diverse views and critiques into a more generalizable formulation, we attempted to augment the power of explanation of the aforementioned traditional stakeholder theory by integrating it with the systems theory [22]. This enabled consideration of the various stakeholder groups from two different viewpoints: “structural”, viewing stakeholders as internal or external to the system, and “functional”, viewing stakeholders as primary or secondary consumers of the system. We aimed to utilize this quadrilateral 2 × 2 matrix of the stakeholders to permit a more comprehensive analysis of the RMT operationalization patterns.

Thirdly, we aimed to develop a statistical formula, representing an operational definition of RMT in Israel that would reflect the dynamic balance between the stakeholder interests and the system’s given standardized goals and empirical constraints and resources [7,18]. This formula, although deriving from the Israeli local case, was expected to offer a generic, empirically based tool for other modern Western countries to determine RMT which coincides with the human rights recommendations endorsed at the Paris UN General Assembly Resolution (United Nations. (n.d.), Universal Declaration of Human Rights, https://www.un.org/en/universal-declaration-human-rights/ (accessed on 4 April 2015)) on 10 December 1948.

### 2.5. Sample Characteristics

This convenience sample comprised 32 Hebrew-speaking informants (18 males; 14 females) with broad relevant knowledge of the study topics [23] who were selected using a purposeful sampling criterion [24] from four different systemic levels of the Israeli health system, as defined by public systems theory [22]. The four stakeholder groups were: (a) regulatory-level stakeholders (*n =* 5; senior Ministry of Health managers/directors); (b) service-level management (*n* = 9; senior managers/directors/administrators of health funds, hospitals, etc.); (c) service-level professionals engaged in direct patient care (*n* = 10; doctors, nurses, secretarial/maintenance staff); (d) consumer-level stakeholders (*n* = 8; citizens/consumers and consumer representatives such as third-sector agents, legal experts, journalists, academicians specializing in public health policy and economics). The participants’ ages were 35–80 years (*M* = ~57). All of them except for two held academic degrees (e.g., lawyers, physicians, accountants, and nurses).

As seen in Table S1 (see Supplementary Materials), the participants were mapped onto a quadrilateral 2 × 2 matrix of primary versus secondary stakeholders belonging to the hardcore internal operation of health service delivery (health-promoting patient-centered care and its delivery subsystems within the national healthcare system) versus the external envelope that determines and supports it (e.g., policy decision makers and civil NGOs). Within the internal and external groups, we divided the stakeholders into primary and secondary ones according to their proximity/distance from the locus of actual medical care delivery and consumption processes. For example, the stakeholders in the primary external group hold a central role in determining the national policies and standards for RMT. The number of stakeholders per level was determined to reflect their weight in and influence on the health system (as asserted in [25]). Thus, the stakeholders in the primary positions in the internal group were in the largest group (*n* = 16), and secondary internal stakeholders were in the smallest group (*n* = 2).

### 2.6. Data Collection

An inductive process was used to collect the personalized, experience-near data [24] via a qualitative interview for the in-depth examination of the medical system stakeholders’ narratives about real situations and concepts in action [26,27]. Individual semi-structured interviews were conducted in Hebrew and audio recorded. The interview questions related to diverse dimensions of the medical system including the socio-economic, ethical-legal, managerial, and clinical aspects of “reasonability” as a standard of responsible action in medicine [12,13]. Based on Fetterman, the interview guide (available from the authors) offered reminders of the key issues that were to be addressed during the interviews [28].

The sample questions are: How would you describe what is currently considered to be RMT in Israel? Is there a gap between your professional/personal expectation for RMT and its existing practice? How would you balance the need to supply sufficiently advanced medical care to highly populated central urban regions versus to remote peripheral regions? How do you feel about economically driven constraints on health funds’ professional prescription of medical treatment? To what extent do you feel that budget-driven medical service is professionally controlled? How do you view the impact and interrelations of the economic allocations to and services offered by the mandatory national medical basket versus the complementary privately offered ones?

The interviews took place in the interviewee’s office (66%) or at their home (25%) or a café (9%) per the interviewee’s request, and it lasted for 46 min on average. To minimize social desirability and comply with the University IRB authorization requirements, we informed the participants of the study’s strict anonymity and confidentiality (with no exposure of their anonymous narratives to their supervisors or senior colleagues and no direct impact on the system and their role in it) and of their study exit options which had no repercussions.

### 2.7. Data Analysis

The content analysis of the interview data comprised several steps. Firstly, to identify the categories, the data were examined by two internal judges (members of the research team, the first and second authors) and one professional external judge (Ph.D. candidate conducting relevant advanced research). All of the judges initially categorized 20% of the data, and once an agreement was achieved, they proceeded to analyze all of the 32 full sets of data. Based on repetitions of pertinent utterances [29], this analysis identified the main categories (e.g., socio-economic, ethical-legal, managerial, and clinical ones), sub-categories (e.g., standards of care), and content categories (e.g., accessibility of care) related to the stakeholders’ perceived, understood, and desired RMT.

Second, the judges searched the individual category-related data for emerging confluence with the main themes. Next, the cross-sectional vertical and horizontal analyses [24] were conducted to check for the interviewees’ positions in relation to the given categories and/or themes, thus, yielding their classification into four systemic stakeholder groups. The initial rule of thumb was that an 80% concurrence among a group’s members would warrant the classification of that group as sharing a category and/or theme. A groups’ concurrence level below 80% was discussed among the judges until they reached an agreement.

### 2.8. Formula Development

Following the stakeholder analysis, we ventured to develop a generic RMT formula that would be simple and universalizable enough, but complex enough, to address the different groups playing roles in each of the system’s operations. We applied current day existing stipulations (see discussion below) so that the formula could serve as a first-approximation indicator to guide the health systems involved in recommending standards for RMT.

## 3. Results

### 3.1. Quadrilateral Stakeholder Analysis

The content analysis yielded one main theme that all of the interviewees raised—“RMT and the healthcare system in Israel”—which concurred with the interview questions’ core topic. As seen in Table S1, the vertical and horizontal analyses of the quadrilateral matrix yielded each stakeholder group’s “viewpoints” (comprising contextual framework, satisfaction level, and target of direct criticism) and “main themes” (comprising focus of commitment, focus of action, and perception of hindrances). On the one hand, all of the participants expressed an overall general satisfaction with the current RMT and healthcare system in terms of its merits (e.g., vision, goals, and agenda) as well as their trust in the system and its continuous improvement. On the other hand, the vast majority of the interviewees (30 out of 32) also offered at least mild criticisms, albeit without specific suggestions for systemic modification. They did identify lacunae in the healthcare system, spanning all of the four aspects of reasonability of care: clinical/service, social/ethical, legal, and economic ones in Table S2 (see Supplementary Materials). The stakeholders’ respective structural position and functional roles in the system were found to determine their evaluation of RMT.

### 3.2. Proposed First-Approximation Statistical Formula for RMT

Based on the Quadrilateral Analytic stakeholder Model analysis outcomes (see Table S1), Equation (1) describes the first-approximation formula which comprehensively reflects the sum of all of the medical services and activities comprising the health system, where different weights ($W_{i}$) ascribed to the n different services are given a generic representation whose values may be specified by future researchers.

(1) $$RMT_{t}=\overset{n}{\underset{i=1}{\sum }}W_{i}\times RMT_{i,t}$$

Equation (2) describes the specific service which is defined as:

(2) $$RMT_{i,t}=CLS_{i,t-1}\times \left(1+\Delta TD_{i,t}\right)\times \left(1+\overset{m}{\underset{j=1}{\sum }}C_{i,t,j}\right)$$

Let us explicate the terms used in the formula. $RMT_{i,t}$ is the ith reasonable medical treatment service at time t, and $CLS_{i,t}$ is the comprehensive level of activity of the ith service (CLS) at time t. Assuming RMT is examined annually, “t” is the current year, while t−1 is the previous year—the basic reference point for the current comprehensive systemic activities and services. These activities and services are reflected in the health basket as a whole: primacy medicine such as tests, outpatient clinics, etc., secondary medicine such as specialists’ clinics, etc., and tertiary medicine such as hospitalization. The additional technological developments ($\Delta TD_{t}=TD_{i,t}-TD_{i,t-1}$) such as the expansion of the healthcare services basket by adding new technologies and the sum of all of the m context variables (C) that influence the ith medical service are added to the basic reference point. C comprises the parameters relating to the services’ accessibility and availability, and its values may be ordinal, interval, or mixed. Or, in other words, these categories add to the abstract constant “C”.

The RMT equation is based on the stakeholders’ variables and their weights. This study focuses on identifying the stakeholders’ variables, which are detailed in Table S2, by distilling them from the cluster of different viewpoints of the stakeholders, which are detailed in Table S1. However, determining the weight of each variable requires additional research to be conducted that would estimate them in the RMT. For the purposes of this study, it was possible to derive the variables’ weights from statistical data and estimate them from different services by utilizing the econometric model.

## 4. Discussion

This qualitative methodology elicited the diverse voices of the Israeli health stakeholders to elucidate the complex health needs of both society as a whole and the individuals in it. Notably, most of the interviewees found the health system in Israel to be reasonably satisfying despite their perceptions of its existing limitations—such as low governmental allocations, budget-driven health-fund practices, and the inferior availability of services in remote geographical locations. Perhaps this pattern of largely positive attitudes may stem from the Israelis’ diachronic and cross-cultural perspectives, that is, the interviewees’ view that Israel’s current system surpasses not only its predecessor but also other systems around the world.

However, these overall positive findings invite researchers to have caution during the interpretation. Israeli citizens are typically accepting of their life constraints and systemic difficulties while maintaining a high level of happiness [30], which is perhaps due to the fact that they are continuously exposed to geopolitical and security threats, which require budgetary and lifestyle accommodations including the funneling of health service budgets to meet existential security needs. Caution in interpreting the current outcomes is further supported by Israel’s overall national capital versus its relatively low healthcare budget (Central Bureau of Statistics, which stated that in 2018, the National Expenditure on Health was 7.6% of the GDP, https://www.cbs.gov.il/en/mediarelease/Pages/2019/In-2018-the-National-Expenditure-on-Health.aspx (accessed on 18 August 2019)) and by frequent media and NGO reports on inaccessible health services and inadequate medical basket decision making in Israel (Ministry of Health, Health policy plan, Jerusalem: Author, 2010) [31]. Thus, the current descriptive data may reflect the Israelis’ pragmatic, acquiescent approach to resource allocation and policy enforcement instead of indicating an optimal healthcare system for rendering RMT.

The purpose of this study was to express the perceptions of the different stakeholders as to how they view “reasonable medical treatment”. By using the qualitative methodology during the analysis of the interviews, we succeeded in arranging the criterions by which a reasonable medical system should be based, according to the outlooks of the stakeholders. A general equation was proposed, and it is one that expresses these criterions. This was the aim of this study: giving an expression for the philosophical question of “what is reasonable medical treatment”. For further research, it would be interesting to understand whether there are different outlooks raised by the stakeholders towards diverse fields within the health system and services. The fields are completely diverse: primary, secondary and tertiary care, each array containing various sub-field, such as preventive care, emergency care, chronic care, and so forth. There is a variety of health services that should be measured, and further research should be conducted developing the suggested equation. As presented, the quantitative outcome of this study, a proposed mathematical equation, may offer a practical tool for different stakeholders in the system (health policy delineators, HMO’s directors, hospital directors, etc.) to use. As mentioned, this tool, as presented at this point, still needs to be developed. In theory, it enables its user to place each and every individual health service in the equation and receive a value that can be then determined if it matches the standard of reasonable health service. The aggregations of all of the scores of the individual health services will result in a comprehensive standard of the health service system as a whole. However, as it has been pointed out, more research has to be conducted in order to use this equation accurately. For example, more research should be conducted in order to determine the weights that each service should receive in the equation. As we have mentioned, the variety of health services is enormous; one cannot refer to all of the services at the same level, i.e., a visit to a clinic is not comparable to heart surgery, and therefore, each service should have its own weight in order to be included properly in the equation. For now, as presented in this study, the equation is general, but it could be used separately in different fields of the health system and different countries: one can measure the health standards concerning, for example, primary care, preventive care, chronic care, and so on, as there are many different fields in the health system. Moreover, the equation contains a global variable “C”, which reflects a variety of parameters, as presented in Table S1. In order to use this equation efficiently, further work should be conducted in order to refine or maybe broaden this constant. Then, a comparison of the standards received for each field of services can be conducted. In this study, we propose the general tool that can be used by the health policy makers, and it can be applied to different fields and levels of care and be adjusted to the different health systems in different countries. By offering a basic practical tool, it may help us understand the needs, and it may be a part of a mechanism that is used by governments to characterize and determine the standard for medical treatment, and it may be a way to measure if this standard is achieved. This is a proposed tool for setting medical policy, then controlling it, and monitoring it.

To conclude, in this research we managed to present the different agenda, outlooks, and visions of the various stakeholders in the system and present them all in a comprehensive practical equation that is to be used by the policy makers. This research lays the foundation for determining the standards for reasonable health services, and it presents a tool that can serve in monitoring and controlling the services offered to civilians. If further research is conducted, referring to different fields of care, there is no doubt that the accuracy and efficiency of the proposed tool will excel, and the novelty of it for the health system will be significant. Even more, in order to standardize the RMT, it would require another study that would simulate the RMT vs. healthcare policies.

In addition, with regard to the present study’s generalizability, one might argue that this proposal rests on findings that are relevant only to Israel’s health system. However, the interview questions elicited diverse stakeholder discourse on many social, ethical, clinical, managerial, financial, and legal issues that could be applicable to other countries. Moreover, Israel offers a scientifically and technologically advanced environment that supports its health system’s efforts to find the optimal level of medical care for all who are within the constraints of the given allocated resources. Thus, the Israeli environment is unique in that it provides researchers and practitioners which represent a convenient pool of candidates for analyzing advanced management practices, serving as a “maduradam” (microcosm) of the developed countries in Western Europe and North America [32].

## 5. Conclusions

While the leaders and experts deal with addressing the direct consequences of the pandemic, less attention is being given to the standards of treatment. This formula will serve as a starting point for determining the standards in times of crisis such as the COVID-19 pandemic. From a practical point of view, the systemic model presented invites a more realistic synergistic approach to healthcare planning and implementation, which accounts for active socio-economic forces and relationships among the regulators (Ministry of Health), the public insurers (health funds), the healthcare providers (physicians, hospitals, etc.), the NGO third-sector consumer representatives, and the consumers. The proposed explicitly formulated measure of RMT may potentially guide decision-making processes in the health system [25,33]. It can be used as a monitoring tool, retrospectively, in order to check whether the policy determined was successful, and it can also be used prospectively as a tool that can be adjusted according to new factors that were not considered from the start, or the change of reality such as that which occurred when COVID-19 entered our lives. Furthermore, it could facilitate more rational and responsible resolutions to balance and integrate the external and internal conflicts among the stakeholders and various representatives of different systemic interests. Moreover, it may permit the critical regulatory assessment and control of the system’s social and health-related effectiveness which are gauged against the social goals [33,34] or even under other political affects [35].

In addition, the proposed scientific formula lends itself to statistical testing for both the validity and effectiveness of the process of evaluating RMT, which can overcome other countries’ scrutiny of its generalizability. Given that the current study’s methodology is solely qualitative, we welcome future researchers’ efforts to critically attempt both the quantitative and qualitative validation of the proposed quadrilateral model for analyzing the stakeholders’ core themes and of the proposed first-approximation statistical tool. Often, qualitative research samples offer small subgroups due to this methodology’s cumbersome nature, yielding different examples and foci in the interviewees’ data [24]. Thus, once it is endorsed judiciously by leaders, the operational definition of RMT and its weighted variables can be determined cross-culturally. Then, this generic formula could become a quantitative instrument with practical applications for the future planning, managing, monitoring, and critical improvement of health systems. We invite research, not only regarding different national health systems, but also systemic comparisons within international mega-systems such as the OECD.

We note that the healthcare administrators’ and policymakers’ conceptualizations of RMT should always remain open to dynamic critical considerations related to medical progress and community development [36]. In particular, the focus should lie on enhancing the leaders’ perceptions of “existing constraints” which are changeable by virtue of a reliable cost/benefit analysis, rather than seeing such constraints as immovable barriers to change [37]. The coefficients of the cost/benefit analyses will be ever-changing as well [22]. Last but not least, the proposed formula and its application in various contexts offers common grounds for the inclusion of ethical and societal considerations into the meta-frameworks tasked with developing the standards for the responsible quality, content, timeliness, and scope of public healthcare.

The goal of this research is to highlight how complex, thus crucial, just policymaking is and how important it is to relate to different standpoints and attitudes concerning the system in order to deliver at the end, a reasonable system with reasonable service and clinical quality standards. Nevertheless, these kinds of changes have consequences. They may affect, for example, issues of control in the system, finance, operations, management, staffing, etc. Specifically, it may result in a budget framework breach, a strike in the power forces and the control that the regulator holds today, a complex interaction of different health sector actors in the system, and there are probably many more aspects to it. Having pointed out the difficulties, further research and discussions ought to follow these questions. Still, a responsible society must make this change and adopt the proposed formula in order to assure reasonable medical treatment for its beneficiaries.

## Supplementary Materials

The following supporting information can be downloaded at: https://www.mdpi.com/article/10.3390/healthcare10122528/s1, Table S1: Study Findings on Healthcare Stakeholders’ Viewpoints and Main Themes by Group Assignment in Quadrilateral Analytic Model (N = 32); Table S2: Summary of Lacunae and their Consequences Described by Interviewees, according to the Four Sub-Categories.

## References

[1] Lethbridge J. (2009). Trade unions, civil society organizations and health reforms. Cap. Cl..

[2] Lor M., Bowers B.J., Jacobs E.A. (2019). Navigating Challenges of Medical Interpreting Standards and Expectations of Patients and Health Care Professionals: The Interpreter Perspective. Qual. Health Res..

[3] White K.L., Williams T.F., Greenberg B.G. (1996). The ecology of medical care. Bull. New York Acad. Med..

[4] Davies S., Winpenny E., Ball S., Fowel T., Rubin J., Nolte E. (2014). For debate: A new wave in public health improvement. Lancet.

[5] Bin Nun G., Berlovitz Y., Shani M. (2005). The Health System in Israel.

[6] Bonnafous-Boucher M., Porcher S. (2010). Towards a stakeholders’ society: Stakeholder theory vs. theory of civil society. Eur. Manag. Rev..

[7] Fottler M.D., Blair J.D. (2002). New concepts in health care stakeholder management theory and practice. Health Care Manag. Rev..

[8] Varvasovszky Z., Brugha R. (2000). Stakeholder analysis: A review. Health Policy Plan..

[9] Duwe E.A.G. (2016). Toward a Story Powerful Enough to Reduce Health Inequities in Indian Country: The Case of Diabetes. Qual. Inq..

[10] Li M. (2009). A harmony of capitalism and socialism?. Sci. Soc..

[11] Sweetbaum H. (2008). Socially responsible capitalism. Soc. Bus. Rev..

[12] Guy D. (2004). Research of the Mental Health Reform in Israel and the Attempts at Its Implementation, 1995–1998. Ph.D. Thesis.

[13] Oxman A.D., Bjorndal A., Becerra-Posada F., Gibson M., Block M.A.G., Haines A., Hamid M., Odom C.H., Lei H., Levin B. (2010). A framework for mandatory impact evaluation to ensure well informed public policy decisions. Lancet.

[14] Jones T.M., Felps W., Bigley G.A. (2007). Ethical theory and stakeholders-related decisions: The role of stakeholder culture. Acad. Manag. Rev..

[15] Freeman R.E. (1994). The politics of stakeholder theory: Some future directions. Bus. Ethics Q..

[16] Clarkson M.B.E. (1995). A stakeholder framework for analyzing and evaluating corporate social performance. Acad. Manag. Rev..

[17] Malvey D., Fottler M.D., Slovensky D.J. (2002). Evaluating stakeholder management performance using a stakeholder report card: The next step in theory and practice. Health Care Manag. Rev..

[18] Parmar L.B., Freeman R.E., Harrison J.S., Wicks A.C., Purnell L., De Colle S. (2010). Stakeholders theory: The state of the art. Acad. Manag. Ann..

[19] Brickson S.L. (2007). Organizational identity orientation: The genesis of the role of the firm and distinct forms of social value. Acad. Manag. Rev..

[20] Eakin M.J. (2016). Educating Critical Qualitative Health Researchers in the Land of the Randomized Controlled Trial. Qual. Inq..

[21] Ford C.R., Henderson J., Handley D.M. (2010). Enhancing long term care for older adults: An exploration of interagency collaboration within geriatric education centers. J. Health Hum. Serv. Adm..

[22] Laor N., Agassi J. (1990). Diagnosis: Philosophical and Medical Perspectives.

[23] Sandelowski M. (1995). Focus on qualitative methods: Sample size in qualitative research. Res. Nurs. Health.

[24] Patton M.Q. (1980). Qualitative Evaluation and Research Methods.

[25] Mainardes E.W., Raposo M., Alves H. (2011). Organizations with dispersed powers: Suggestion of a new management model based on the stakeholders theory. J. Manag. Res..

[26] Morse J.M. (2003). The Significance of Standards. Qual. Health Res..

[27] Guba E.G., Lincoln Y.S. (1989). Fourth Generation Evaluation.

[28] Fetterman D.M. (1989). Ethnography: Step by Step.

[29] Strauss A., Corbin J. (1990). Basics of Qualitative Research: Grounded Theory Procedures and Techniques.

[30] Helliwell J.F., Layard R., Sachs J.D. *World Happiness Report 2019*. United Nations Sustainable Development Solutions Network, 2019. https://worldhappiness.report/ed/2019/.

[31] Reiter V. (2010). The Pattern of Relationship between Trust and Collaboration amongst Non-Profit Organizations and Health Funds.

[32] Harel G.H., Tzafrir S.S. (1999). The effect of human resource management practices on the perceptions of organizational and market performance of the firm. Hum. Resour. Manag..

[33] Severin F., Borry P., Cornel M.C., Daniels N., Fellmann F., Hodgson S.V., Howard H., John J., Kaariainen H., Kayserili H. (2015). Points to consider for prioritizing clinical genetic testing services: A European consensus process oriented at accountability for reasonableness. Eur. J. Hum. Genet..

[34] Maheshwari A.K., Werd M.R.P., Travis F., Rainforth M., Lipman J. (2022). Workplace well-being: An experimental investigation into benefits of consciousness-based architecture. J. Manag. Spiritual. Relig..

[35] Nisani D. (2018). Efficient indirect regulation under protection for sale. J. Regul. Econ..

[36] Johns G. (2006). The essential impact of context on organizational behavior. Acad. Manag. Rev..

[37] Shani S., Siebzehner M.I., Luxenburg O., Shemer J. (2000). Setting priorities for the adoption of health technologies on a national level: The Israeli experience. Health Policy.

