# Supplementary material for: A New Tool for Determining and Monitoring Public Healthcare Systems
Source: Healthcare (Basel). 2022 Dec 14;10(12):2528. doi: 10.3390/healthcare10122528 (PMC9777851; doi:10.3390/healthcare10122528)
Supplement: Supplementary file 1 [file healthcare-10-02528-s001.zip › healthcare-2055780-supplementary.pdf]

Table S1:

Study Findings on Healthcare Stakeholders' Viewpoints and Main Themes by Group Assignment in Quadrilateral Analytic Model (*N *= 32)

**PRIMARY-LEVEL GROUPS**		**STAKEHOLDER POSITION VIS-À-VIS HEALTH ORGANIZATIONS**	
		**PRIMARY INTERNAL GROUP (*n* = 16)**	**PRIMARY EXTERNAL GROUP (*n* = 6)**
		**Field medical staff (*n* = 7); Senior medical/administrative directors from health funds, hospitals, etc. (*n* = 9)**	**Ministry of Health policymakers and directors (*n* = 3); Ordinary citizens (*n* = 3)**
**Group viewpoints**	*Satisfaction level*	Overall satisfaction with universal medical system and treatment offered	Overall satisfaction with universal medical system and treatment offered
	Target of direct criticism	Concrete - Criticism toward own system's employees and managers	Abstract - Criticism of medical system's service provision to society as a whole and/or to individuals
	Contextual framework	Availability, accessibility (e.g., location, distance, waiting list) and socio-cultural considerations	Availability, accessibility (e.g., location, distance, waiting list) and socio-cultural considerations
**Main themes**	*Focus of commitment*	Patient care – Staff stands for patients (fights inequity) within system's limitations	System's regulation – Decision makers (e.g., Ministry of Health) involved with system control and monitoring
	Focus of action	Solutions for intra-systemic conflictual goals;<br>Understanding and operating successfully in system's hierarchy/restrictions – Being aware of Ministry of Health's sometimes conflicting, arbitrary, patient-indifferent demands	Offering standard of care within systemic constraints;<br>Perception of National Health Insurance Law as mostly positive
	Perception of hindrances	Budget shortages and misallocations;<br>Plethora of regulative control	Overly bureaucratic processes;<br>Still facilitating inequity due to the need for costly supplementary insurance

		STAKEHOLDER POSITION VIS-À-VIS HEALTH ORGANIZATIONS (Continued)	
**SECONDARY-LEVEL GROUPS**		**SECONDARY INTERNAL GROUP (*n* = 2)**	**SECONDARY EXTERNAL GROUP (*n* = 8)**
		**Administrative and maintenance workers serving consumers (*n* = 2)**	**Members of 1994-5 reform committee (senior directors emeritus from Ministry of Health or health funds; *n* = 3); Representatives of patients' organizations (*n* = 3); Public representatives (*n* = 2)**
**Viewpoints**	*Satisfaction level*	Overall satisfaction with universal medical system and treatment offered	Overall satisfaction with universal medical system and treatment offered
	Target of direct criticism	Limited focus on own level of functioning.<br>Lack of interest in the health system as a whole	Severe criticism leveled primarily at public representatives, perceiving Ministry of Health as not proactive on socio-political and related medical economic matters
	Contextual framework	Availability and accessibility considerations	Availability, accessibility (e.g., location, distance, waiting list) and socio-cultural considerations
**Main themes**	*Focus of commitment*	Patient care – Concern with quality of medical care	Quality of equal optimal universal healthcare for all citizens
	Focus of action	Fulfilling one’s job requirements	Monitoring and criticism of public health care policy and its implementation.<br>Identifying systemic conflicts of interest (e.g., Ministry of Health both owns and regulates hospitals; health funds both mediate insurance and provide ambulatory and hospital services to citizens)
	Perception of hindrances	Budget shortages and misallocations	Ministry of Health policymakers’ failure to (a) recognize the whole system’s complexity (raised particularly by public representatives), and (b) allocate national budgets given principles of distributive justice and welfare, resulting in social inequity and damage to medical professionalism

Table S2: ***Summary of Lacunae and their Consequences Described by Interviewees, according to the Four Sub-Categories***

Reasonability sub-category	Lacunae	Possible consequences
**Economic**	1. Public share of national health expenditure is greatly drained by the private sector	• Socio-economic gaps lead to inequality
	2. Some central health services have not been included in the National Health Insurance Law basket	• Interests of consumer groups impact on all-too-central health policy considerations and budget allocation
	3. Budget is controlled and determined by the Ministry of Finance, which is, at times, arbitrary	• Harm to entire sub-populations
	4. Ministry of Health is not assertive and decisive enough in representing health needs of the population	• Lack of funding for healthcare-related resources (personnel and technology) – of necessity, affecting level of treatment<br>• Inability to engage in long-term systemic planning
	5. Lack of control, supervision, and budgetary updates regarding financial aspects in the system	• Exceeding budget allocations
**Clinical/ service**	6. Insufficient time allocated to the doctor/patient session	• Lower standard of treatment<br>• Off-hand superfluous referrals to tests and specialist examinations (burnout of professionals and patients) as well as unnecessary incurred expenses
	7. Long waiting list	• Hindrance to medical treatment and service<br>• Coping via lowering of standard of care
	8. Paucity of measurements for service, clinical controls, and monitoring	• Lower level of treatment<br>• Underbalanced expenses
	9. Defensive medicine	• Tension between the regulator (Ministry of Health) and operators (health funds/hospitals/medical insurance companies)
	10. Ministry of Health's guidelines are often formulated in an inapplicable/ambiguous manner	• Confusion concerning protocols and standards on part of health providers as well as consumers' expectations
	11. Ministry of Health is not aware of the concrete needs of the citizens and the system	• Insufficient and inappropriate treatment
	12. Frustration of personnel in the system	• Unpleasant work environment

**Legal**	13. The term "reasonable" is vaguely formulated in the National Health Insurance Law	• State absolved from commitment<br>• No treatment standardization (boundaries, quality)<br>• Ambiguity allows socio-economic status to determine access to optimal treatment, resulting in hopelessness and retreat from treatment in weaker groups
	14. System is bureaucratic, cumbersome, and inflexible	• Lack of service and treatment
	15. Health basket should be expanded	• For better medical treatment
	16. Ministry of Health holds conflicting dual responsibilities: policy planner and regulator as well as operator of medical services	• Biased allocations and self-monitoring harms distributional social justice and systemic ethics as well as public transparency
**Social/ ethical**	17. Lack of services in rural areas	• Inequality in the health system
	18. Discrimination between populations	
	19. Lack of human and technological resources	• Less patient/doctor session time → reduced level of care<br>• Physician procedures transferred to nurses' responsibility
	20. High co-payment required from citizens	• Unequal service availability and accessibility<br>• Social injustice<br>• Low socio-economic population pushed to refrain from consuming necessary health services
	21. System is not transparent: Patients are not aware of their medical rights	• Reduced systemic commitment to service quality<br>• Lack of medical treatment
	22. Supplementary insurance	• Increases inequality between populations<br>• Increases the national health expenses
	23. Senior management officials often overvalue administrative considerations over professional and moral ones	• Deterioration of human and professional standards in the practice of medicine
